# Increased lanosterol turnover: a metabolic burden for daunorubicin-resistant leukemia cells

**DOI:** 10.1007/s12032-015-0717-5

**Published:** 2015-12-23

**Authors:** Claudia Stäubert, Rosanna Krakowsky, Hasanuzzaman Bhuiyan, Barbara Witek, Anna Lindahl, Oliver Broom, Anders Nordström

**Affiliations:** Department of Molecular Biology, Umeå University, 90187 Umeå, Sweden; Department of Forest Genetics and Plant Physiology, Swedish Metabolomics Centre, Swedish University of Agricultural Sciences, Umeå, Sweden; Doping Laboratory, Department of Clinical Pharmacology, Karolinska University Hospital, Stockholm, Sweden; Department of Oncology-Pathology, Science for Life Laboratory, Karolinska Institutet, Stockholm, Sweden; Institute of Biochemistry, Faculty of Medicine, University of Leipzig, Leipzig, Germany

**Keywords:** Leukemia, Drug resistance, Cholesterol biosynthesis, LC–MS, Stable isotope labelling mass spectrometry, Cancer

## Abstract

The cholesterol metabolism is essential for cancer cell proliferation. We found the expression of genes involved in the cholesterol biosynthesis pathway up-regulated in the daunorubicin-resistant leukemia cell line CEM/R2, which is a daughter cell line to the leukemia cell line CCRF-CEM (CEM). Cellular ^2^H_2_O labelling, mass spectrometry, and isotopomer analysis revealed an increase in lanosterol synthesis which was not accompanied by an increase in cholesterol flux or pool size in CEM/R2 cells. Exogenous addition of lanosterol had a negative effect on CEM/R2 and a positive effect on sensitive CEM cell viability. Treatment of CEM and CEM/R2 cells with cholesterol biosynthesis inhibitors acting on the enzymes squalene epoxidase and lanosterol synthase, both also involved in the 24,25-epoxycholesterol shunt pathway, revealed a connection of this pathway to lanosterol turnover. Our data highlight that an increased lanosterol flux poses a metabolic weakness of resistant cells that potentially could be therapeutically exploited.

**Electronic supplementary material:**

The online version of this article (doi:10.1007/s12032-015-0717-5) contains supplementary material, which is available to authorized users.

## Introduction

Cholesterol is an essential component of mammalian cell membranes and serves as a precursor for bile acids and various endocrine steroid hormones. The biosynthesis, cellular absorption, and efflux of cholesterol are tightly regulated to maintain homeostatic levels required for normal cell proliferation. A link between cholesterol and cancer was proposed over a century ago, with the discovery that tumor cells had accumulated cholesterol [1]. Since then, many studies have provided evidence for a link between carcinogenesis/tumor progression and cholesterol biosynthesis and efflux [2]. Elevated activity of hydroxymethylglutaryl-coenzyme A reductase (HMGCR), the first enzyme of the mevalonate pathway, has been shown in a range of different tumors including hepatocellular carcinoma [3], leukemia [4] and lymphoma [5]. Moreover, inhibition of HMGCR, the initial and rate-limiting step of cholesterol biosynthesis, with statins inhibits tumor growth in mouse xenograft models [6–8]. Epidemiological data further support a role for statins in reducing the risk of developing pancreatic cancer [9] and with an increased progression-free survival in inflammatory breast cancer [10].

Cancer is a clonal disease whereby therapeutic intervention poses a selective pressure resulting in cancer cells escaping therapy. Surviving cells are characterized by drug resistance and are often associated with disease relapse [11]. Several reports support a function of cholesterol in establishing and maintaining increased drug tolerance in cancer cells. Cholesterol has been found to be increased by 50 % in isolated plasma membranes of vinblastine-resistant versus sensitive acute lymphoblastic leukemia cells (ALL) [12]. The observation that drug-resistant myeloid leukemia cell lines are more sensitive to statins than their sensitive parental lines further substantiates a role for cholesterol in chemoresistance [13]. Moreover, in vitro treatment of acute myeloid leukemia (AML) cells with chemo- or radiotherapy causes increased intracellular cholesterol levels accompanied by an increased drug tolerance, whereas inhibition of cholesterol biosynthesis with statins could restore drug sensitivity [14]. Further, it has been shown that rat and human hepatocellular carcinoma cells display increased mitochondrial cholesterol levels and HMGCR or squalene synthase (FDFT1) inhibition sensitizes those cells to mitochondria-directed chemotherapy [15]. Similarly, in a doxorubicin-resistant bladder cancer cell line, simultaneous administration of statin with doxorubicin reverted the resistant phenotype [16].

We have recently shown that resistance to daunorubicin (DNR) in an ALL cell line is associated with a rewired metabolism [17]. RNA Sequencing revealed the cholesterol biosynthetic pathway as the top canonical pathway up-regulated in the resistant cells [17].

In the present study, we validate these previous findings using quantitative real-time PCR (RT-qPCR) and measure relative quantity and synthesis rates of cholesterol itself and lanosterol, the first committed intermediate in cholesterol biosynthesis, by application of ^2^H_2_O labelling and mass spectrometry isotopomer analysis. We found that the transcriptional up-regulation of the cholesterol biosynthesis pathway does not translate into an increased cholesterol synthesis rate or quantity in the resistant cells, but rather an increased flux through the lanosterol pool. With this report we shift the focus from the importance of solely cholesterol for cancer progression and drug sensitivity to the upstream biosynthetic intermediate lanosterol. Our data reveal a previously unrecognized metabolic cost of cancer drug resistance and point toward a potential novel regulatory role of lanosterol in maintaining cholesterol homeostasis, which may be particularly critical for drug-resistant leukemia cancer cells.

## Materials and methods

### Cell lines and growth conditions

CCRF-CEM [CCRF CEM] (ATCC^®^ CCL-119™) (CEM) leukemia cells were acquired through LGC Standards (Teddington, UK) from the American Type Culture Collection and maintained following the recommendations from ATCC. Detailed description of the generation of the DNR-resistant CEM/R2 is described in [17].

### Proliferation assays: ATPlite™ (Perkin Elmer)

Cells were seeded in black plates with a final density of 15,000 cells/well. Simultaneously, the respective treatment was started. ATPlite™ was performed following the manufacturer’s instructions. Cells were incubated for 48 h with or without cholesterol biosynthesis inhibitors, namely, atorvastatin (100 µM), terbinafine (25 µM), ketoconazole (20 μM), triparanol (2 µM), CI976 (25 µM), all purchased from Sigma-Aldrich (MO, USA), and hymeglusin (10 µM), YM-53601 (10 µM) and BIBB-515 (25 µM) all ordered from Santa Cruz Biotechnology (TX, USA) in the absence (vehicle control DMSO or MeOH) or presence of DNR (CEM, 1 nM and CEM/R2, 0.5 μM) in RPMI 1640 (HyClone, Fisher scientific) supplemented with 10 % FBS.

Lanosterol, cholesterol, and 1,2-dimyristoyl-sn-glycero-3-phosphocholine (PC), all obtained from Sigma-Aldrich (MO, USA), were dissolved in chloroform/methanol (1:1). Cholesterol or lanosterol was mixed in equimolar proportion with PC and dried by vacuum in a speed vacuum concentrator. The lanosterol/PC, cholesterol/PC mixture, or PC alone was re-suspended in serum-free RPMI 1640 on the day of the experiment and used within the day of preparation.

To analyze the effect of lanosterol and cholesterol, cells were incubated in serum-free RPMI 1640 medium (HyClone, Fisher scientific) for 48 h in the absence or presence of DNR (CEM, 100 nM and CEM/R2, 1 μM). The negative/vehicle control always contained respective amounts of DMSO, MeOH, or PC.

### RNA isolation, reverse transcription, and quantitative real-time PCR

One million cells of each CEM and CEM/R2 cells were seeded in a 6-w plate and cultured for 24 h in RPMI supplemented with 10 % FBS without or with 50 µM atorvastatin, 12.5 µM terbinafine, 12.5 µM BIBB515, or 10 µM ketoconazole before harvested for RNA preparation. Total RNA isolation was performed using RNeasy^®^ Mini Kit (Qiagen, Germany) following manufacturer’s instructions. RNA (1 µg) was treated with DnaseI (NEB) prior reverse transcription using iScript™ cDNA Synthesis Kit (Bio-Rad) following the manufacturer’s instructions. The qPCR was set up using iTaq™ Universal SYBR^®^ Green Supermix (Bio-Rad), and real-time PCR and data collection were performed on Bio-Rad^®^iQ™5 Real-Time PCR Detection System. Primer design procedure and detailed description of each step can be found in [18]. All qPCR primer pairs are stated in Table S1. Expression of gene-encoding proteins involved in cholesterol biosynthesis was normalized to the reference genes RPL13A, RPS18, ACTB, and GAPDH.

### Liquid chromatography–mass spectrometry (LC–MS) measurements

For experiments with ^2^H_2_O, 1 × 10^6^ cells were cultured for 4 h (without or with 50 µM atorvastatin, 12.5 µM terbinafine, 12.5 µM BIBB515 or 10 µM ketoconazole) or 24 h (untreated comparison of lanosterol and cholesterol turnover in CEM versus CEM/R2), in RPMI 1640 medium supplemented with 10 % FBS which was either diluted with sterile H_2_O or ^2^H_2_O (99 %) to a final concentration of 30 % ^2^H_2_O (Cambridge Isotope Laboratories (MA, USA)).

The cells were centrifuged, washed at least once with PBS, transferred to an 1.5-mL Eppendorf tube and lysed/extracted using 200 μL 50:50 chloroform/methanol to which a small lab spoon of 0.2 μm i.d. glass beads was added (Retsch). Tubes were placed in a Retsch Beadmill MM 400 and shaken at 30 Hz for 2 min. Eppendorf tubes were transferred to a centrifuge kept at 4 °C and spun at 14,000 rpm for 10 min after which the supernatant was transferred to LC–MS glass vials, dried down in a speed vacuum concentrator, and stored at −20 °C until analysis. Samples were dissolved in 20 μL chloroform out of which 2–4 μL were injected into the Agilent 1290 LC system connected to either a 6540 or 6550 Agilent Q-TOF mass spectrometer (CA, USA) and an atmospheric pressure ionization (APCI) source was used. Data were collected between m/z 70 and 1700 in positive ion mode only. The following APCI settings were used: gas temperature 200 °C, vaporizer 350 °C, gas flow 11 l/min, nebulizer pressure 40 psig, Vcap 3500, corona 4, fragmentor 100, Skimmer1 45, and OctapoleRFPeak 750. All samples were separated using reverse phase only, Kinetex C18, 100 mm × 2.1 mm, 2.6 μM 100 Å, Phenomenex (CA, USA). For elution, solvents reversed phase (A) H_2_O, 0.1 % formic acid (B) 75:25 methanol/isopropanol, 0.1 % formic acid were used. All solvents were of HPLC grade. Linear gradients were used for all separations and were devised as follows for reversed phase separation (0.5 mL/min) min 0: 5 %B, min 8: 95 %B, min 10: 95 %B, min 10.2: 5 %B, min 12: 5 %B. Raw data were processed and analyzed using MassHunter Qual, Agilent (CA, USA). Identification of metabolites in all experiments was carried out using synthetic standards obtained from Sigma-Aldrich (MO, USA) and Inventia Pty. Ltd (NSW, Australia) comparing accurate mass, retention time, and in some cases MS/MS spectra.

## Results

### The cholesterol biosynthetic pathway is up-regulated in DNR-resistant CEM/R2 cells

**Fig. 1:**
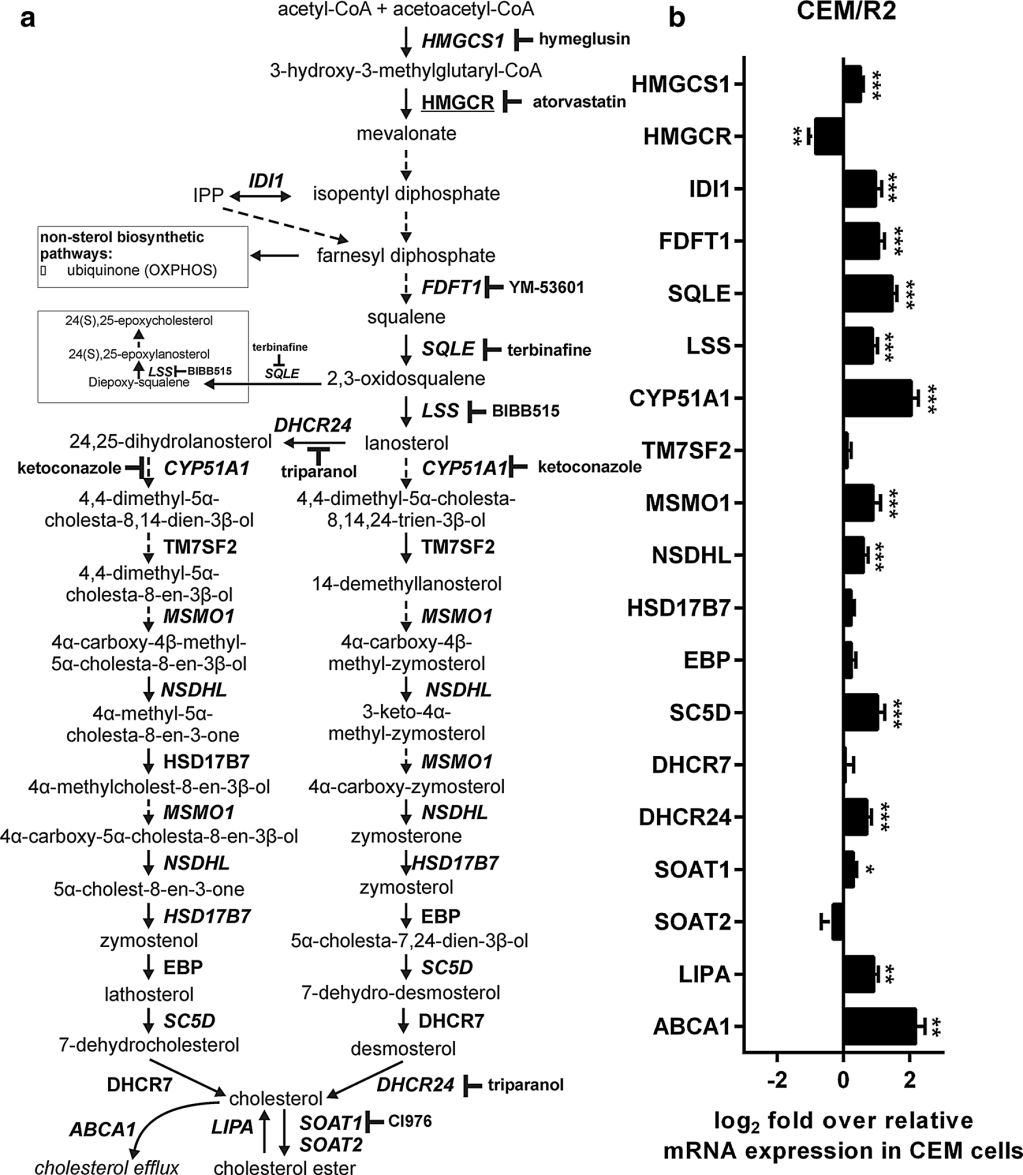
Cholesterol biosynthesis pathway is altered in CEM/R2 cells. **a** Outlined cholesterol synthesis pathway in which all genes with increased mRNA expression are highlighted in *green* and HMGCR as its mRNA expression level is lower in CEM/R2 cells is highlighted in *red*. Major points of inhibition by cholesterol biosynthesis inhibitors are highlighted in *red.* **b** mRNA expression levels of all genes involved in the cholesterol biosynthetic pathway is shown as log2 fold over relative mRNA expression for each gene in CEM cells. Significance was assessed using a two-tailed unpaired *t* test. Data are shown as mean ± SEM of six independent experiments. *P ≤ 0.05; **P ≤ 0.01; ***P ≤ 0.001

Levels of gene expression for all cholesterol biosynthetic genes in CEM and CEM/R2 were monitored using RT-qPCR (Fig. 1a, b). Interestingly, mRNA levels of HMGCR are significantly lower in CEM/R2 cells (Fig. 1b), whereas mRNA levels of all other enzymes involved in cholesterol biosynthesis except five are significantly up-regulated (Fig. 1b). The highest fold change in mRNA expression was obtained for CYP51A1 (cytochrome P450, family 51, subfamily A, polypeptide 1) and ABCA1 with approximately fivefold higher mRNA levels in CEM/R2 when compared to CEM cells followed by squalene epoxidase (SQLE) with approximately threefold higher mRNA levels in CEM/R2 cells (Fig. 1b).

### Increased flux through the lanosterol but not the cholesterol pool in resistant CEM/R2 cells

**Fig. 2:**
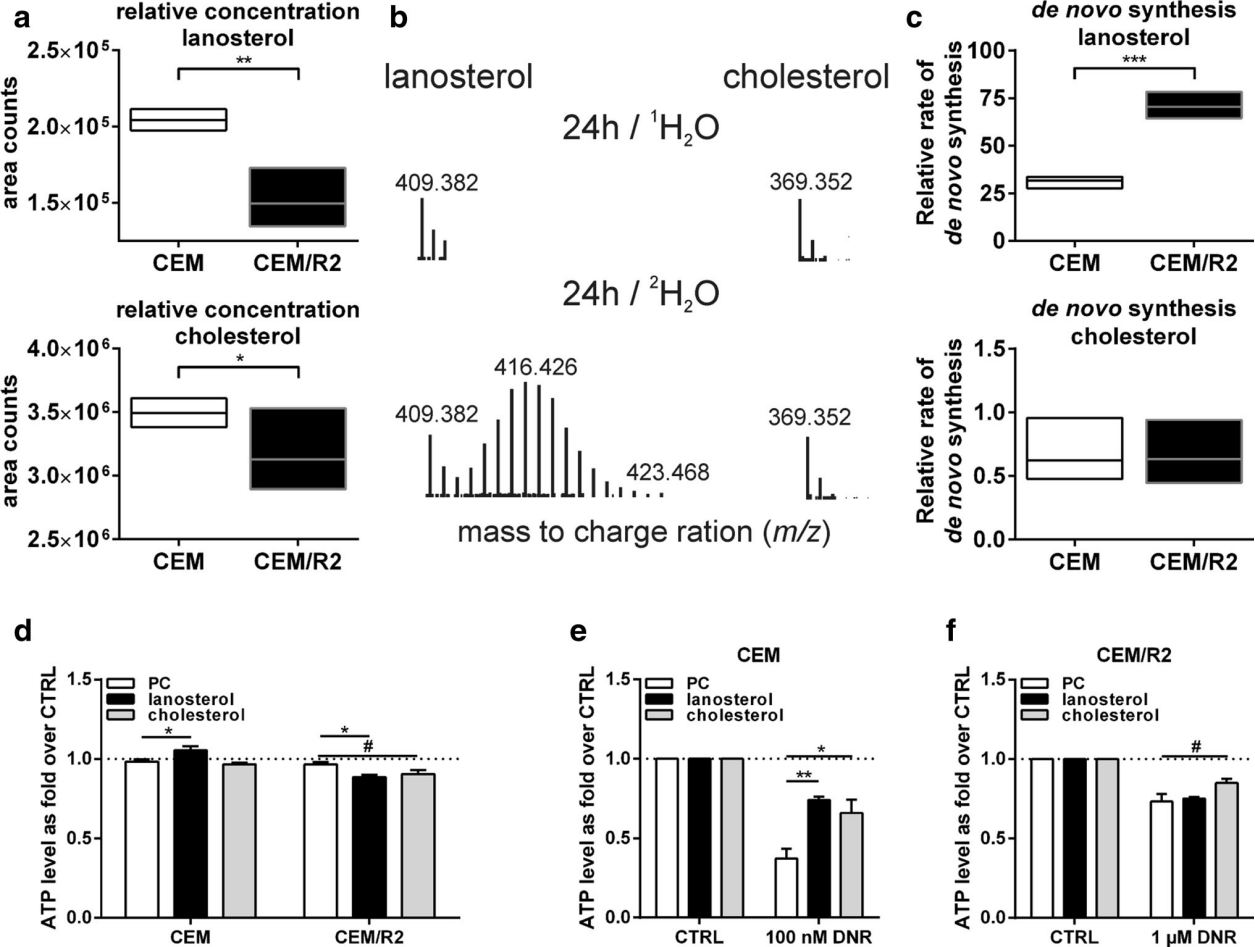
Resistant leukemia cells CEM/R2 exhibit an increased flux through the lanosterol but not the cholesterol pool and are negatively affected by exogenous lanosterol addition. **a** Relative concentration of lanosterol and cholesterol in CEM versus CEM/R2 cells as determined by LC–MS. **b** Mass spectra of lanosterol (*left*) and cholesterol (*right*) after cell growth for 24 h using regular media (*top*) and media with addition of 30 % ^2^H_2_O (*bottom*). **c** Data showing de novo synthesis of lanosterol and cholesterol measured on cells grown in 30 % ^2^H_2_O. **a**–**c** Data of a single experiment carried out in five replicates are shown as minimum to maximum with line at mean. **d** Viability of CEM and CEM/R2 cells (*n* = 4) that were grown for 48 h in serum-free RPMI 1640 in presence of 50 μM 1,2-dimyristoyl-sn-glycero-3-phosphocholine (PC), lanosterol/PC mixture (each 25 μM), or cholesterol/PC mixture (each 25 μM). Viability of CEM (**e**) and CEM/R2 (**f**) cells that were incubated for 48 h in absence or presence of DNR (CEM, 100 nM and CEM/R2, 1 μM) and 50 pM PC, lanosterol/PC mixture (each 25 μM), or cholesterol/PC mixture (each 25 μM). **d**–**f** Data are shown as mean ± SEM of four independent experiments carried out in triplicate, *P* values were determined using an ordinary one-way ANOVA with Dunnett’s multiple comparisons test. ^#^≤0.1; *P ≤ 0.05; **P ≤ 0.0110

Next, we measured relative quantity of lanosterol, the first cholesterol biosynthesis intermediate committed solely to the cholesterol pathway, and cholesterol using liquid chromatography–mass spectrometry (LC–MS). Surprisingly, we found that in spite of a transcriptionally up-regulated cholesterol pathway in CEM/R2 cells, the relative concentration of lanosterol and cholesterol was lower in CEM/R2 cells (Fig. 2a). This observation led us to analyze whether the lower relative quantity of lanosterol and cholesterol can be explained by lower synthesis rate in CEM/R2 cells. Thus, we set out to measure de novo synthesis of cholesterol itself and lanosterol using ^2^H_2_O labelling and LC–MS [19]. By growing cells in media diluted with ^2^H_2_O, the stable isotope ^2^H will be incorporated throughout the cellular metabolism which can be followed in the individual metabolites by mass spectrometry and isotopomer analysis, illustrated in Fig. 2b. Virtually, no de novo formation of cholesterol could be observed (Fig. 2b, c). Lanosterol on the other hand which displayed a lower relative concentration in CEM/R2 cells compared to CEM (Fig. 2a) exhibited at the same time a higher relative ^2^H incorporation in CEM/R2 cells, suggesting a higher flux through the lanosterol pool in the resistant cells (Fig. 2c).

### Exogenous addition of lanosterol is beneficial for drug-sensitive CEM but disadvantageous for resistant CEM/R2 cells

With no apparent transfer of the ^2^H label from lanosterol to cholesterol (Fig. 2b), it is reasonable to assume that in CEM/R2 cells the increased lanosterol production reflects that lanosterol, rather than just being an intermediate in the cholesterol biosynthesis either is exported out of the cells or fills another function. Thus, to probe whether increased lanosterol flux is essential to maintain resistance or rather a metabolic consequence of resistance, we investigated the influence of exogenous addition of lanosterol and cholesterol on CEM and CEM/R2 cell viability (Fig. 2d). Lanosterol addition was beneficial for CEM but disadvantageous for CEM/R2 cell viability (Fig. 2d). Cholesterol had no apparent effect on CEM cells and was slightly disadvantageous for CEM/R2 cells (Fig. 2d). Next, we evaluated the pro-survival effect of lanosterol and cholesterol on DNR sensitivity of CEM and CEM/R2 cells (Fig. 2e, f). Presence of both lanosterol and cholesterol decreased the sensitivity of CEM cells to DNR (Fig. 2e), thus supporting a pro-survival effect of lanosterol for cancer cells. However, in CEM/R2 cells, exogenous lanosterol addition did not trigger such an effect and cholesterol addition decreased sensitivity to DNR only slightly (Fig. 2f). We conclude therefore that the increased lanosterol flux represents a metabolic cost rather than a survival advantage for the resistant CEM/R2 cells.

### Differential sensitivity of CEM versus CEM/R2 cells toward cholesterol biosynthesis inhibitors

**Fig. 3:**
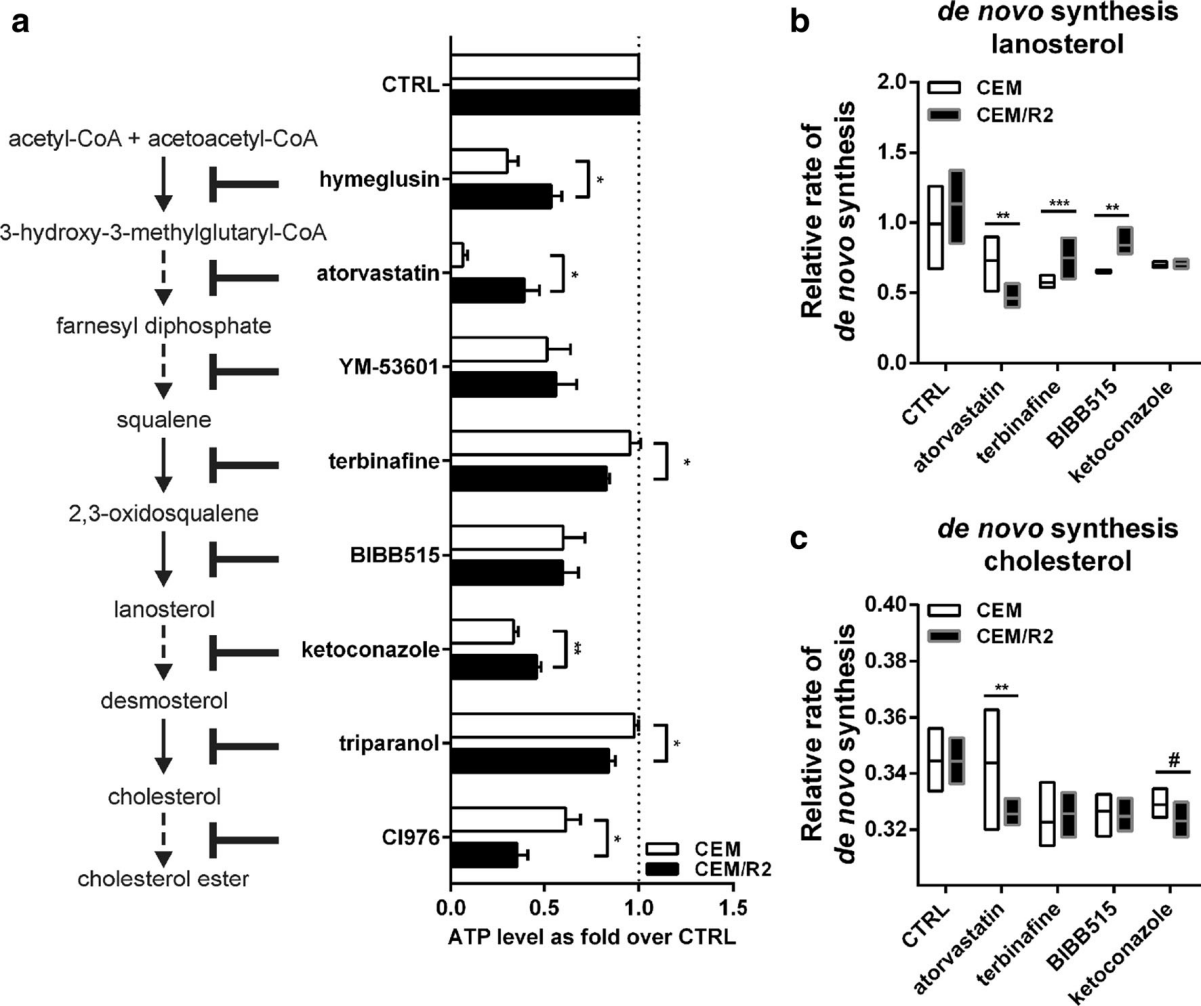
Effect of cholesterol biosynthesis inhibitors on cell viability and lanosterol as well as cholesterol relative synthesis rate of CEM and CEM/R2 cells. **a** CEM and CEM/R2 cells were incubated for 48 h with 10 μM hymeglusin, 100 μM atorvastatin, 10 μM YM-53601, 25 μM terbinafine, 25 μM BIBB-515,10 μM ketoconazole, 2 μM triparanol, or 25 μM CI976 in RPMI 1640 supplemented with 10 % FBS in comparison with vehicle control DMSO or MeOH. Hymeglusin, atorvastatin, and ketoconazole had a stronger effect on CEM cells, whereas terbinafine, triparanol, and CI976 were more effective on CEM/R2 cells, and no difference in sensitivity between CEM and CEM/R2 cells was observed for YM-53601 and BIBB-515. Data are shown as mean ± SEM of three independent experiments carried out in triplicates. Relative synthesis rate of lanosterol (**b**) and cholesterol (**c**) in CEM and CEM/R2 cells as determined by LC–MS after cells were cultured for 4 h in presence or absence of 50 μM atorvastatin or 12.5 μM terbinafine, 12.5 μM BIBB515 or 10 μM ketoconazole, in RPMI 1640 supplemented with 10 % FBS and 30 % ^2^H_2_O. Data of a single experiment carried out in four replicates are shown as minimum to maximum with line at mean. **a**–**c** Significance was assessed using a two-tailed unpaired *t* test ^#^≤0.1; *P ≤ 0.05; **P ≤ 0.01; ***P ≤ 0.001

To gain further insights into the differences of the cholesterol biosynthetic pathway between sensitive and resistant leukemia cells, with special focus on both rate-limiting steps and steps producing or consuming lanosterol, we evaluated the potential of different cholesterol biosynthesis inhibitors as both cytostatic agents (Fig. 3a) and positive modulators of drug sensitivity (Figure S1) in CEM and CEM/R2 cells.

If cholesterol lowering *per se* has an anticancer effect, the transcriptional up-regulation of the cholesterol biosynthesis pathway in CEM/R2 cells points toward an increased relevance of this pathway in resistant cells. Therefore, one would expect that all inhibitors of cholesterol biosynthesis, administered alone, would have a stronger effect on CEM/R2 cell viability. This did not turn out to be the case. However, terbinafine, an antifungal compound inhibiting SQLE, triparanol, an inhibitor of 24-dehydrocholesterol reductase (DHCR24), both shown to suppress tumor growth [20–23], as well as CI976, a potent and selective Acyl-CoA/cholesterol acyltransferase (SOAT1) inhibitor [24, 25] did affect CEM/R2 more than the sensitive CEM cells (Fig. 2a).

### Lanosterol de novo synthesis rate correlates with sensitivity toward cholesterol biosynthesis inhibitors

If the increased lanosterol flux is a metabolic burden for the resistant cells, while being associated with increased viability in sensitive cells, one could argue that cholesterol synthesis inhibitors inducing a relatively larger reduction in lanosterol flux in resistant compared to sensitive cells should result in lower viability of sensitive CEM to CEM/R2 cells or vice versa. To test this hypothesis, we analyzed the effect on de novo synthesis of lanosterol and cholesterol when exposed to atorvastatin acting at HMGCR, the first rate-limiting enzyme, terbinafine, acting at the proposed second rate-limiting step SQLE, BIBB-515, which inhibits lanosterol synthase (LSS), as well as ketoconazole, an inhibitor of the CYP51A1 [26, 27] directly downstream of lanosterol (Fig. 3b, c). All four inhibitors reduced the de novo synthesis rate of lanosterol and cholesterol (except of atorvastatin in CEM cells) significantly in both cell lines when compared to control (Fig. 3b, c). Next, we compared the relative degree of reduction of lanosterol de novo synthesis for sensitive versus resistant cells. We observed that atorvastatin reduced the de novo synthesis through the lanosterol pool more in resistant cells, whereas the opposite was true for terbinafine and BIBB515 (Fig. 3b). No difference in lanosterol biosynthesis rate comparing CEM and CEM/R2 cells was observed when cells were treated with ketoconazole (Fig. 3b). The comparison of the viability data obtained for atorvastatin and terbinafine (Fig. 3a) with the lanosterol flux information gained by LC–MS (Fig. 3b) reveals a connection between sensitivity to those inhibitors and their effect on lanosterol de novo synthesis rate.

### Inhibition of the 24,25-epoxycholesterol shunt pathway suggests its differential regulation in resistant cells

**Fig. 4:**
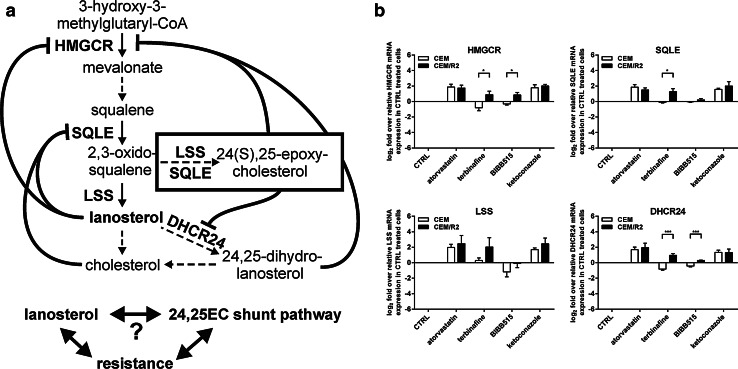
Differentially regulated 24,25-EC shunt pathway in CEM/R2 cells potentially cause or effect of increased lanosterol flux. **a** HMGCR, the first rate-limiting step of cholesterol biosynthesis is negatively regulated by lanosterol, 24,25-dihydrolanosterol and 24,25-EC [50]. SQLE is regulated by lanosterol and cholesterol [41–45] and participates, together with LSS, in a shunt of the mevalonate pathway that produces 24,25-EC. 24,25-EC itself inhibits HMGCR [46] and DHCR24 [47]. **b** BIBB515 and terbinafine, acting at LSS and SQLE, respectively, induce differential expression of HMGCR, SQLE, and DHCR between CEM and CEM/R2. Cells were cultured for 24 h in presence or absence of 50 μM atorvastatin, 12.5 μM terbinafine, 12.5 μM BIBB515, or 10 μM ketoconazole in RPMI 1640 supplemented with 10 % FBS. mRNA expression levels are shown as log2 relative mRNA expression fold over vehicle-treated CEM or CEM/R2 cells, respectively. Data are shown as mean ± SEM of four independent experiments, *P* values were determined using an unpaired *t* test. *P ≤ 0.05; **P ≤ 0.01; ***P ≤ 0.001

The resistant cells show an increased flux of lanosterol which is disadvantageous for them. It is reasonable to assume that these cells have regulatory mechanisms that are different when compared to sensitive cells. These mechanisms can either be the cause of or are required to manage the observed increased lanosterol flux. One such regulatory mechanism of cholesterol biosynthesis is the 24,25-epoxycholesterol (24,25-EC) shunt pathway (Figs. 1a, 4a). 24,25-EC has been shown to cause a decreased cholesterol synthesis rate by inhibition of HMGCR [28] and DHCR24 [29] and thus prevents accumulation of newly synthesized cholesterol which could cause ER stress and cell toxicity [30, 31]. Since the inhibitors of this shunt pathway, namely terbinafine inhibiting SQLE and BIBB515 inhibiting LSS, had both reduced de novo synthesis of lanosterol more in CEM/R2 cells, we evaluated their effect on mRNA level of genes involved in the cholesterol biosynthetic pathway, in comparison with the effects induced by atorvastatin and ketoconazole treatment (Fig. 4b, Figure S2).

Both BIBB515 and terbinafine but not atorvastatin and ketoconazole induce differential expression of HMGCR, SQLE, and DHCR24 when comparing sensitive and resistant CEM cells (Fig. 4b). Several other genes that are involved in the main cholesterol biosynthesis pathway display the same pattern (Figure S2). The inhibition of the 24,25-EC shunt pathway, but not the main cholesterol biosynthesis pathway, produces an up-regulation of rate-limiting steps of the cholesterol biosynthetic pathway specific to resistant cells (Fig. 4b). We are therefore suggesting that either the shunt pathway is critical for resistant cells to regulate an increased lanosterol flux or a differentially regulated shunt pathway is the cause for the increased lanosterol flux.

## Discussion

An altered cholesterol metabolism is critical for rapidly proliferating cancer cells and plays a role in development of resistance. Based on our findings, we suggest that lanosterol, the first intermediate committed solely toward cholesterol biosynthesis, is a valuable marker to detect alterations of the cellular cholesterol homeostasis. We show that the increased lanosterol flux in a DNR-resistant daughter cell line of the T-ALL leukemia cell line CEM represents a metabolic cost that can potentially have therapeutic implications. In a broader sense, our results highlight that phenotyping cancers with respect to cholesterol metabolism can be useful for therapy guidance.

We used RT-qPCR and compared mRNA levels of all proteins involved in the cholesterol biosynthesis pathway (Fig. 1a) between DNR-sensitive CEM cells and the resistant daughter cell line CEM/R2. The almost uniformly increased expression in the resistant cells (Fig. 1b) was not matched by increased levels of cholesterol or lanosterol (Fig. 2a). Using ^2^H_2_O labelling of cultured cells, we could demonstrate an increased biosynthetic flux of lanosterol (Fig. 2b, c) with no concomitant accumulation of lanosterol or cholesterol (Fig. 2a). It is therefore reasonable to assume that in CEM/R2 cells the increased lanosterol production reflects that lanosterol, rather than just being an intermediate in the cholesterol biosynthesis, is exported out of the cell [32–34] or fills another function. Membrane associated lanosterol will alter plasma membrane organization relative to cholesterol [35], which potentially could impact drug tolerance [12]. Further, lanosterol has been proposed to act as a survival factor for dopaminergic neurons, potentially via a mitochondrial decoupling mechanism [36]. We observed that exogenously applied lanosterol negatively affected viability of resistant cell but had a positive effect on cell viability of DNR-sensitive CEM cells. Moreover, since lanosterol has been described as a survival factor [36], we tested the effect of lanosterol on DNR sensitivity of both CEM and CEM/R2 by co-administration of DNR and lanosterol, which revealed that lanosterol presence decreased DNR sensitivity of CEM cells but had no such effect on CEM/R2 cells. We conclude from the results from all those experiments that the increased lanosterol flux is a stressor for the resistant cells and thus a negative consequence of the resistance. Consequently, it is reasonable to hypothesize that resistant cells have different regulatory mechanisms of the cholesterol biosynthetic pathway to cope with the increased lanosterol flux. Inhibition of different steps of the cholesterol pathway revealed differential effects on viability when comparing sensitive and resistant cells (Fig. 3a). Further, using ^2^H_2_O labelling mass spectrometry, we show that inhibitors that reduce the lanosterol synthesis rate more in the resistant cells affected their viability less upon treatment with the respective inhibitors (Fig. 3b, c).

Lanosterol itself has been shown to be involved in posttranslational regulation of the cholesterol biosynthesis pathway through induction of proteasomal degradation of HMGCR [37] and SQLE [38]. The enzyme SQLE, which is inhibited by terbinafine, is further transcriptionally [39, 40] and posttranslationally regulated by cholesterol [41, 42] and participates, together with LSS, which can be inhibited by BIBB515, in a shunt of the mevalonate pathway that produces 24,25-EC (Figs. 1a, 4a). By measuring gene expression of all genes in the cholesterol biosynthesis when applying one of the inhibitors, atorvastatin, terbinafine, BIBB515, or ketoconazole, a pattern emerged. Inhibitors of SQLE or LSS, both of which are enzymes involved in the main pathway and in the 24,25-EC shunt pathway, resulted in a reciprocal expression pattern for many genes when comparing CEM versus CEM/R2. In contrast, atorvastatin and ketoconazole that do not affect the 24,25-EC shunt pathway exert the same effects on mRNA levels of cholesterol biosynthesis genes in both sensitive and resistant cells (Figs. 4b, S2).

In conclusion, our data provide a novel connection between drug resistance and increased lanosterol flux and also links the 24,25-EC shunt pathway with resistance. We believe that there is a high potential for exploitation of this knowledge in personalized therapy guidance.

## Electronic supplementary material

Supplementary material 1 (DOC 799 kb)

## Abbreviations

ALL: Acute lymphoblastic leukemia
CoA: Coenzyme A
DNR: Daunorubicin
FBS: Fetal bovine serum
LC–MS: Liquid chromatography–mass spectrometry
P-gp: P-glycoprotein
