# Supplementary material for: Increased lanosterol turnover: a metabolic burden for daunorubicin-resistant leukemia cells
Source: Med Oncol. 2015 Dec 23;33:6. doi: 10.1007/s12032-015-0717-5 (PMC4689760; doi:10.1007/s12032-015-0717-5)
Supplement: Supplementary file 1 — Supplementary material 1 (DOC 799 kb) [file 12032_2015_717_MOESM1_ESM.doc]

# *Supplementary material*

# Increased lanosterol turnover - a metabolic burden for daunorubicin resistant leukaemia cells

Claudia Stäubert^1,2,5^, Rosanna Krakowsky^5^, Hasanuzzaman Bhuiyan^3^, Barbara Witek^1^, Anna Lindahl^4^, Oliver Broom^1^ and Anders Nordström^1,2,4#^

Running Title: Resistance in leukaemia cells and lanosterol

^1^ Department of Molecular Biology, Umeå University, Umeå, Sweden

^2^ Swedish Metabolomics Centre, Department of Forest Genetics and Plant Physiology, Swedish University of Agricultural Sciences, Umeå, Sweden

^3^ Doping Laboratory, Department of Clinical Pharmacology Karolinska University Hospital

Stockholm, Sweden

^4^ Department of Oncology-Pathology, Science for Life Laboratory, Karolinska Institutet, Stockholm, Sweden

^5^ Institute of Biochemistry, Faculty of Medicine, University of Leipzig, Leipzig, Germany

^#^Address correspondence to: Anders Nordström, Department of molecular biology, SE-90187, Umeå, Sweden, Phone: +46 90 785 25 61; Fax: 090-77 26 30; E-mail: [anders.nordstrom@umu.se](mailto:anders.nordstrom@umu.se)

**Figure S1**


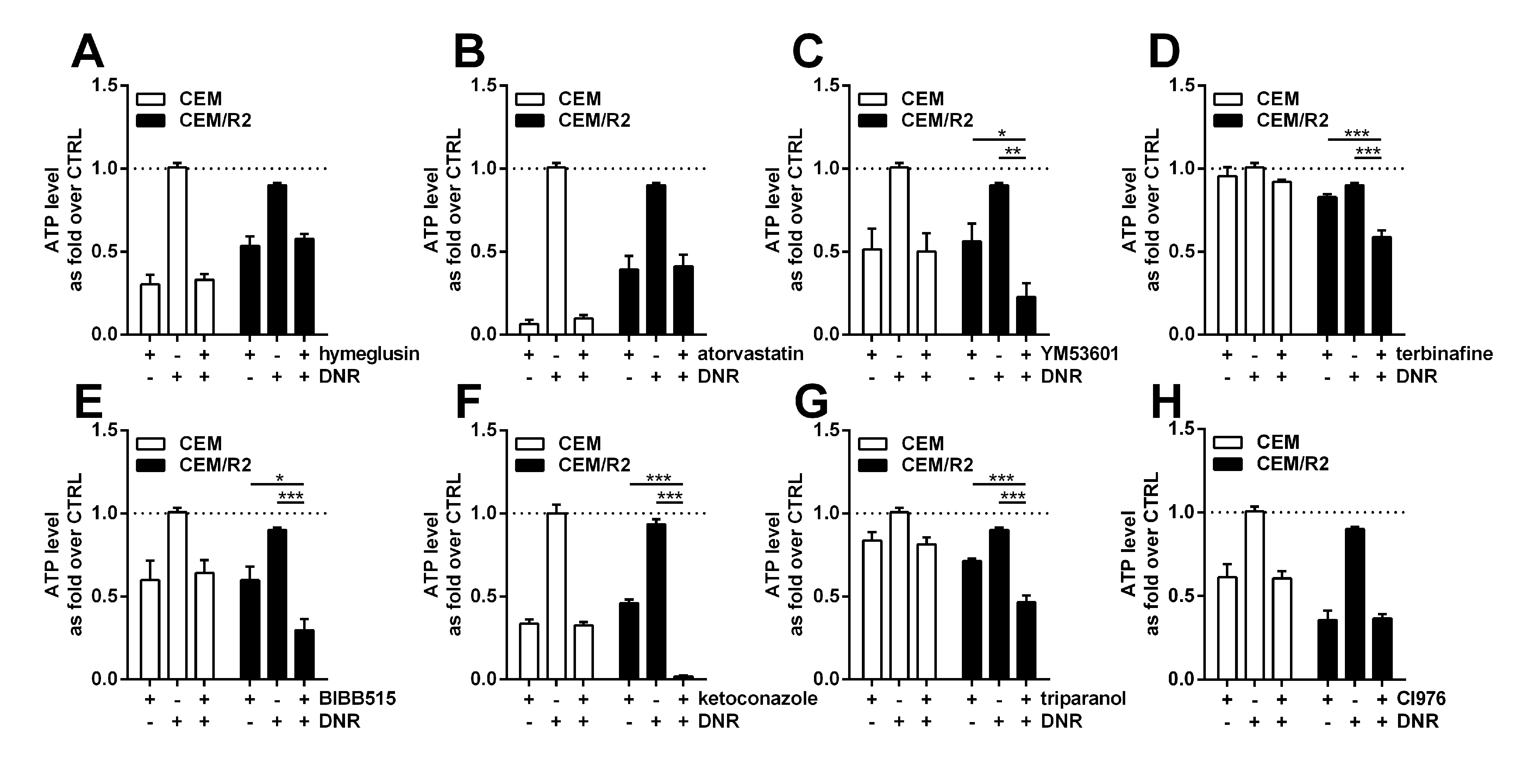


**Figure S1 Sensitizing effect of co-administration of cholesterol biosynthesis inhibitors and DNR on CEM and CEM/R2 cell viability.** Co-administration of cholesterol biosynthesis inhibitors (A) 10 µM hymeglusin, (B) 100 µM atorvastatin, (C) 10 µM YM-53601, (D) 25 µM terbinafine, (E) 25 µM BIBB-515, (F) 20 µM ketoconazole, (G) 2 µM triparanol, (H) 25 µM CI976, for 48h in RPMI1640 supplemented with 10% FBS, acting down-stream of HMGCR except CI976, sensitized CEM/R2 cells to an otherwise non-lethal concentration of DNR (0.5 µM) whereas no sensitizing effect was observed for co-administration of any inhibitor and 1 nM DNR in CEM cells. Significance was assessed using a two-tailed unpaired t test. Data is shown as mean ± SEM of 3 independent experiments. * P ≤ 0.05; ** P ≤ 0.01; *** P ≤ 0.001.

**Figure S2**

**
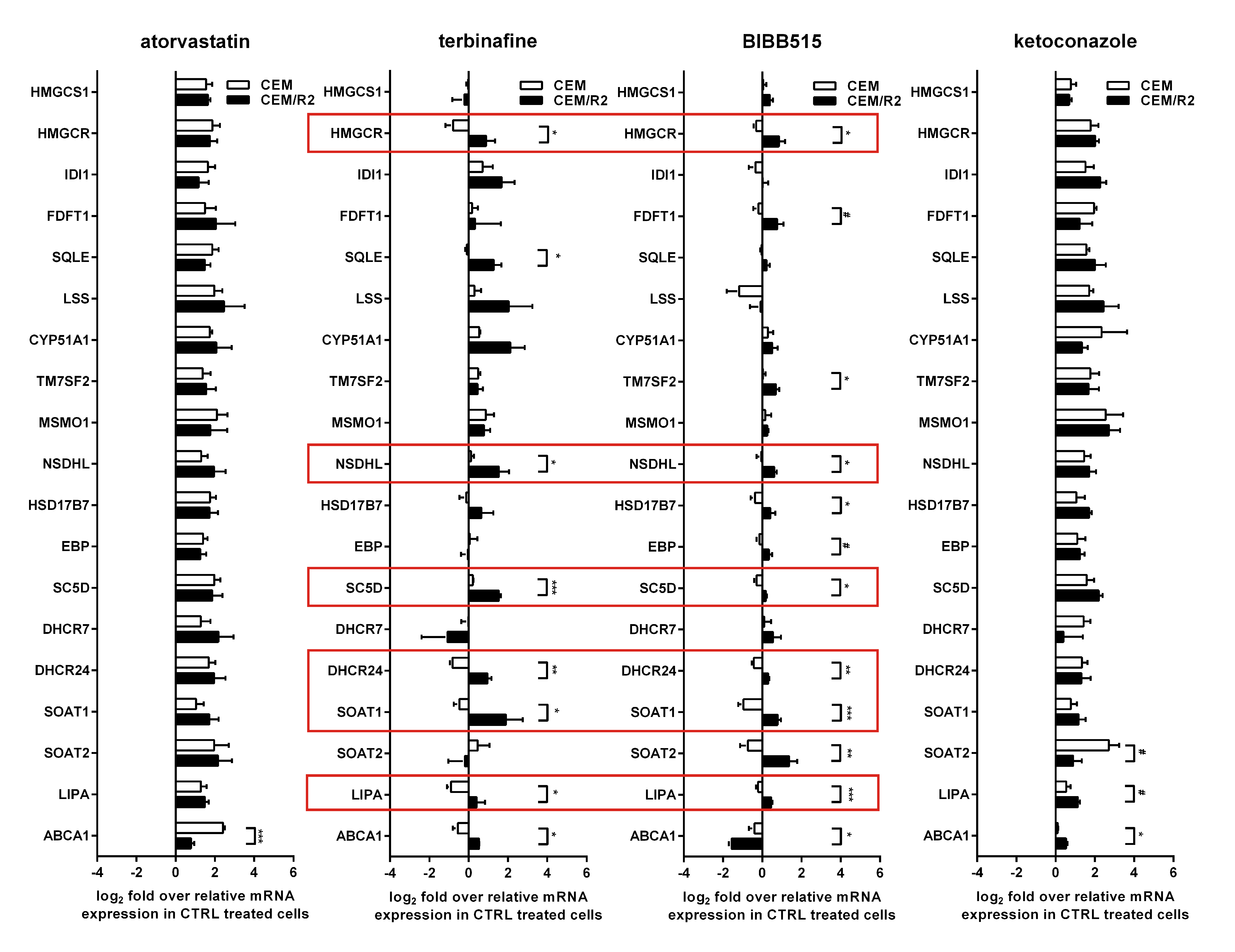
**

**Figure S2 Changes in mRNA expression levels of genes involved in the cholesterol biosynthesis pathway upon atorvastatin, terbinafine, BIBB515 or ketoconazole treatment of CEM and CEM/R2 cells.** mRNA expression levels of all genes involved in the cholesterol biosynthetic pathway is shown as log_2_ fold over relative mRNA expression for each gene in control (DMSO or MeOH) treated CEM or CEM/R2 cells, respectively. CEM and CEM/R2 cells were cultured for 24h in presence or absence of 50 µM atorvastatin, 12.5 µM terbinafine, 12.5 µM BIBB515 or 10 µM ketoconazole in RPMI1640 supplemented with 10% FBS. Data is shown as mean ± SEM of 4 independent experiments. p-values were determined using a two-tailed unpaired t test. ^#^ ≤ 0.1; * P ≤ 0.05; ** P ≤ 0.01; *** P ≤ 0.001.

**Table S1 qPCR primer sequences**

| **Name** | **Sequence (5’-3’)** |
| --- | --- |
| RPS18-106-forward | GATGGGCGGCGGAAAATAG |
| RPS18-192-reverse | GTCTGCTTTCCTCAACACCAC |
| ACTB-1412-forward | ACAATGTGGCCGAGGACTTT |
| ACTB-1519-reverse | TGGGGTGGCTTTTAGGATGG |
| RPL13A-179-forward | GTCGTACGCTGTGAAGGCAT |
| RPL13A-279-reverse | CGGGAAGGGTTGGTGTTCAT |
| GAPDH-121-forward | CAGTCAGCCGCATCTTCTTTTG |
| GAPDH-206-reverse | AATCCGTTGACTCCGACCTTC |
| HMGCS1-50-S | CGGAGTCGGGTGGGTTG |
| HMGCS1-179-AS | TCACTGTTTCCTCCTTCGGG |
| HMGCR-1909-S | GCAGATGGGATGACTCGTGG |
| HMGCR-2031-AS | AGTGCTGTCAAATGCCTCCT |
| IDI1-292-S | GCCTGAAATAAACACTAACCACCT |
| IDI1-435-AS | AATCCTTTCTCAATGTTCTCGTTCA |
| FDFT1-475-S | GGCAGGAGCCACCGAG |
| FDFT1-614-AS | TGGGGACTGGAGGTGAGG |
| SQLE-974-S | GTTCGGGGACTTCATCACTTTG |
| SQLE-1089-AS | CGAGGAGACCCCCGTTTC |
| DHCR24-1064-S | TGAAGACAAACCGAGAGGGC |
| DHCR24-1180-AS | CAGCCAAAGAGGTAGCGGAA |
| DHCR7-284-S | AATCGCAACCCAACATTCCC |
| DHCR7-395-AS | GCCAGTGAAAACCAGTCCAC |
| LSS-1507-S | GCACATCCCCAGAGAACGG |
| LSS-1600-AS | ACGCTTGGTCTCATAGGTGG |
| MSMO1-763-S | TCTTCTTTGGGCATGGGTGA |
| MSMO1-879-AS | TGATGCCGAGAACCAGCATA |
| CYP51A1-651-S | AGTTTCAGACGCAGGGACAG |
| CYP51A1-790-AS | GGACGCCCATCCTTGTATGT |
| SC5D-365-S | GTCCGTCGAGAGATTAAGTTTACTG |
| SC5D-514-AS | ACTAACGACAAGTTCAAACAATCCA |
| NSDHL-139-S | CGCCTACGGACGGAAAAGAA |
| NSDHL-234-AS | CGACTTGGTCTCTCATTGGCT |
| SOAT1-388-S | AAGTTGACAGCAGAGGCAGAG |
| SOAT1-513-AS | GAGAGCGCACCCACCATT |
| SOAT2-407-S | CGCAAGTCCCTGCTTGATGA |
| SOAT2-506-AS | CCAGGGTGCTGATGATGAAGA |
| HSD17B7-499-S | GGACTTCAGGAGGTGTTTGAGA |
| HSD17B7-598-S | TGAGCTGAGATGGATTGTCACT |
| TM7SF2-928-S | GGCTTCATGCTGGCGTTTG |
| TM7SF2-1077-AS | ACGGAAGATGTAGTAACCAGTAGC |
| EBP-519-S | CCAAGGGAGACAGCCGATAC |
| EBP-654-AS | AGAATGAAGCGGAGGGGATG |
| ABCA1-7127-S | AGTGGACGTTGCAGTTCTCA |
| ABCA1-7218-AS | TTCAGCCACCCCGTATGAAC |
